# Nilgai antelope display no signs of infection upon experimental challenge with a virulent *Babesia bovis* strain

**DOI:** 10.1186/s13071-024-06316-3

**Published:** 2024-06-01

**Authors:** Tammi L. Johnson, Kelly A. Persinger, Naomi S. Taus, Sara K. Davis, Karen C. Poh, Lowell S. Kappmeyer, Jacob M. Laughery, Janaína Capelli-Peixoto, Kimberly H. Lohmeyer, Massaro W. Ueti, Pia U. Olafson

**Affiliations:** 1 Texas A&M AgriLife Research, Uvalde, TX 78801 USA; 2 Department of Rangeland, Wildlife, and Fisheries Management, Texas A&M University, College Station, TX 77843 USA; 3 USDA-ARS, Animal Disease Research Unit, Washington State University, Pullman, WA 99164 USA; 4 Department of Veterinary Microbiology and Pathology, Washington State University, Pullman, WA 99164 USA; 5 Livestock Arthropod Pests Research Unit, Knipling-Bushland United States Livestock Insects Research Laboratory, USDA-ARS, Kerrville, TX 78028 USA

**Keywords:** Bovine babesiosis, *Rhipicephalus* (*Boophilus*) *microplus*, Wildlife host, *Boselaphus tragocamelus*, *Babesia bovis*

## Abstract

**Background:**

Bovine babesiosis is caused by infection with the protozoal parasite *Babesia bovis*, which is transmitted by *Rhipicephalus* (*Boophilus*) spp. It can cause mortality rates up to 90% in immunologically naive *Bos taurus* cattle. In south Texas, *R*. (*B*.) *microplus* is known to infest nilgai antelope (*Boselaphus tragocamelus*); however, their susceptibility to infection with *B*. *bovis* and their role in the transmission of the parasite remain unknown. In this study, we challenged nilgai antelope with *B*. *bovis* and evaluated their susceptibility to infection.

**Methods:**

Nilgai were needle inoculated with ≈10^8^ *B*. *bovis*-parasitized erythrocytes (merozoites) or a homogenate of *B*. *bovis*-infected larval ticks (sporozoite) delivered intravenously. *Bos taurus* beef calves were inoculated in parallel, as this strain of *B*. *bovis* is lethal to cattle. Temperature and hematocrit were monitored daily over the course of each study, and whole blood was collected for molecular [polymerase chain reaction (PCR)] and serological [indirect enzyme-linked immunosorbent assay (ELISA)] diagnostic evaluation. Histological sections of nilgai cerebral tissue were examined for evidence of infection. Recipient bovine calves were sub-inoculated with blood from nilgai challenged with either stage of the parasite, and they were monitored for clinical signs of infection and evaluated by a PCR diagnostic assay. Red blood cells (RBCs) from prechallenged nilgai and *B*. *taurus* beef cattle were cultured with an in vitro *B*. *bovis* merozoite culture to examine colonization of the RBCs by the parasite.

**Results:**

Nilgai did not display clinical signs of infection upon inoculation with either the merozoite or sporozoite stage of *B*. *bovis*. All nilgai were PCR-negative for the parasite, and they did not develop antibodies to *B*. *bovis*. No evidence of infection was detected in histological sections of nilgai tissues, and in vitro culture analysis indicated that the nilgai RBCs were not colonized by *B*. *bovis* merozoites. Cattle subinoculated with blood from challenged nilgai did not display clinical signs of infection, and they were PCR-negative up to 45 days after transfer.

**Conclusions:**

Nilgai do not appear to be susceptible to infection with a strain of *B*. *bovis* that is lethal to cattle. Tick control on these alternative hosts remains a critical priority, especially given their potential to disseminate ticks over long distances.

**Graphical Abstract:**

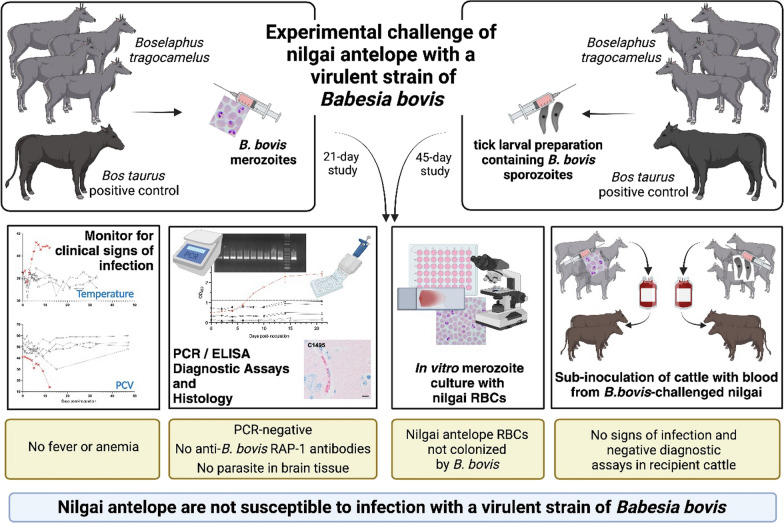

**Supplementary Information:**

The online version contains supplementary material available at 10.1186/s13071-024-06316-3.

## Background

Disease outbreaks in the agricultural industry have a significant economic impact owing to production losses associated with animal deaths, the cost of disease eradication efforts, and the potential effects on trade-related regulations [1]. A major disease outbreak in the US cattle herd, a primary commodity, would have a substantial effect on both cattle producers and consumers. Of importance is the reemergence of bovine babesiosis, a major threat to the health of the beef cattle industry. Bovine babesiosis is caused by infection with either of two species of *Babesia* protozoa parasites (*Babesia bigemina* or *Babesia bovis*), and it was widespread within the USA in the mid-1800s, killing millions of cattle and devastating the cattle industry [2, 3]. The disease was considered eradicated from the USA by 1943, primarily via the elimination of disease vector populations, namely the cattle fever ticks (*Rhipicephalus* (*Boophilus*) *microplus* and *Rhipicephalus* (*Boophilus*) *annulatus*. Eradication was achieved largely by efforts of the Cattle Fever Tick Eradication Program (CFTEP) implemented by the US Department of Agriculture (USDA) in cooperation with the Texas Animal Health Commission [4].

While the USA remains free of bovine babesiosis, incursions of the tick vectors are consistently documented in the Texas–Mexico transboundary region and pose an ever-present threat to the potential reemergence of the disease [5]. Texas is home to the largest number of beef cattle in the USA, with an estimated 12.5 million head, representing more than 13% of cattle in the USA [6]. The reemergence of bovine babesiosis in Texas would impose strict quarantines on cattle moving out of the state and would be devastating to not only Texas cattle ranchers but also the entire US beef industry, including feedlot operations, abattoirs, and retailers. Preventing bovine babesiosis is a national problem, and the role of wildlife species in disease recurrence is not understood.

Tick control on cattle is efficient and highly effective at eliminating ticks; however, the shifting economy and landscape in south Texas toward hunting [7] has resulted in large populations of native and nonnative species of wild ungulates, i.e., white-tailed deer (*Odocoileus virginianus*), nilgai antelope (*Boselaphus tragocamelus*), red deer (*Cervus elaphus*), etc. These species are viable alternative hosts for cattle fever ticks, as the ticks can reproduce on these hosts [8–10], and various life stages of the tick have been recovered from them [11, 12]. As such, they provide a dispersal mechanism for ticks, as they can travel large distances and occupy areas previously free of cattle fever ticks [13–15]. Further, wildlife can readily cross the border between Mexico and the USA [16]. Treatment of these hosts to reduce cattle fever tick infestations is problematic and challenging [17]. The abundance of these ungulate hosts has drastically transformed the ecology of this disease system since it was eradicated in the mid-1900s.

It is critical to understand the potential involvement of wildlife species in the transmission of parasites, specifically the ability of exotic wildlife to serve as reservoirs for *Babesia* spp. White-tailed deer and nilgai antelope were reported to be polymerase chain reaction (PCR)-positive and seropositive for *B*. *bovis* and *B*. *bigemina* [18–22]. However, these findings do not definitively demonstrate that the *Babesia* parasites established infection in the mammalian host or that the animals can mount a parasitemia high enough to infect naïve ticks. In fact, white-tailed deer failed to become infected/infectious when challenged with a virulent strain of *B*. *bovis*, indicating that these hosts are not a reservoir [23]. Similar studies in other ungulates that are populous at the Texas–Mexico transboundary region are essential. In this study, we evaluated the susceptibility of nilgai antelope to infection with a virulent strain of *B*. *bovis* to examine the role of this exotic wildlife species in the *B*. *bovis* transmission cycle.

## Methods

### Study animals

Nilgai calves ranging in age from approximately 2–6 weeks, based on body weight, were wild captured and transferred to the AgriLife Research Center in Uvalde, Texas, where they were bottle raised and acclimated to human interaction. Beef calves (*Bos taurus*), approximately 4 months old, were used as positive control animals, as they are highly susceptible to infection with *B*. *bovis*. All animal procedures were approved by Texas A&M AgriLife Animal Care and Use Committee (#2019–018A), the Texas A&M Institutional Animal Care and Use Committee (#2019–0401), and the Institutional Animal Care and Use Committee of the University of Idaho, Moscow, Idaho (#2021–72). Two experimental groups, each comprised of four nilgai calves and one beef calf, were used in this study. Group 1 was inoculated with *B*. *bovis* merozoites (blood stabilate), and Group 2 was inoculated with a *B*. *bovis* larval tick sporozoite preparation.

### *Babesia bovis* inoculum

#### Blood stabilates

*Babesia bovis* S74-T3Bo DMSO stabilates were generated from infected blood passed through a splenectomized beef calf and cryopreserved in liquid nitrogen as previously described [24, 25]. Nilgai (4–6 months old) were inoculated intravenously with *B*. *bovis* S74-T3Bo stabilates. A bovine steer was similarly inoculated as a positive control. Each animal received a 2 ml intravenous inoculation consisting of 1 ml *B*. *bovis* stabilate (~10^8^ infected erythrocytes) with 1 ml of Puck’s Saline G plus 10% recipient serum.

#### Larval sporozoite preparation

*R.* (*B.*) *microplus* larvae were applied under a cloth patch on a splenectomized, tick-naïve beef calf. At 12 days after larval infestation, *B*. *bovis* S74-T3Bo stabilate (10^7^) was inoculated intravenously into the calf, and replete female ticks were collected during an ascending parasitemia. Egg masses from infected female ticks were pooled and incubated at 26 °C, 96% relative humidity. After hatching, larvae from infected females were placed under a cloth patch on a naïve calf to stimulate the development of *B*. *bovis* sporozoites as previously described [26, 27]. Partially fed larvae were forcibly removed after 4 days of feeding, and infected larvae were incubated on ice for 30 min. Approximately 2000 larvae were added to cold, sterile phosphate buffered saline (PBS; pH 7.4) and ground using a tissue homogenizer. Homogenates were pooled and centrifuged at 400 × *g* at 4 °C for 10 min to recover *B*. *bovis* sporozoites, and the final volume was raised to 6 ml with PBS. To challenge the nilgai and positive control beef calf, the animals were inoculated intravenously with a 2 ml mixture consisting of 1 ml of supernatant containing *B*. *bovis* from infected larvae and 1 ml of Puck’s Saline G plus 10% recipient serum.

### Clinical and molecular detection of *Babesia bovis* infection

Rectal temperature and packed cell volume (PCV) were monitored daily beginning 1 day postinoculation (dpi) for clinical signs of disease. Nilgai whole blood samples and sera were collected daily for the first 10 days, and at 12, 14, and 21 dpi (blood stabilate experiment) or daily for 7 days and at 9, 12, 15, 18, 21, 24, 27, 30, 33, and 47 dpi (larval sporozoite experiment). One animal in the larval sporozoite experiment (N011) suffered a leg injury at 5 dpi and was only subsequently sampled at 6, 15, and 47 dpi. The DNeasy Blood and Tissue Kit (Qiagen, Germantown, MD) was used to extract genomic DNA from whole blood, and the DNAs were used as template in a nested PCR targeting 18S ribosomal RNA (rRNA) [28]. The 18S rRNA outer primers (Nbab-1 forward, 5′-AAG CCA TGC ATG TCT AAG TAT AAG CTT TT-3′ and Nbab-1 reverse, 5′-CTT CTC CTT CCT TTA AGT GAT AAG GTT CAC-3′) were used in a primary PCR with the following conditions: 95 °C for 5 min; 35 cycles of 95 °C for 30 s, 50 °C for 30 s, and 72 °C for 1 min; and final extension at 72 °C for 5 min. The reaction was conducted in 20 μl containing 2 μl of DNA (equivalent to 1 μl whole blood), 2.0 mM MgCl_2_, 200 μM each dNTP, 1.0 μM each primer, and 1 U of Platinum^™^ Taq Polymerase (Thermo Fisher Scientific, Waltham, MS). The 18S rRNA nested primers (forward, 5′- AATCCTGACACAGGGAGGTAGTGAC-3′ and reverse, 5′-CTAAGAATTTCACCTCTGACAGT-3′) amplify a diagnostic fragment of 390 bp. Nested PCR was carried out with the following conditions: 95 °C for 5 min; 35 cycles of 95 °C for 30 s, 65 °C for 30 s, and 72 °C for 30 s; and final extension at 72 °C for 5 min. The reaction was conducted in 20 μl containing 0.1 μl from the first reaction, 2.0 mM MgCl2, 200 μM each dNTP, 1.0 μM of each primer, and 1 U of Platinum^™^ Taq Polymerase. DNA isolated from all collection dates was screened by PCR, and 10 replicate amplifications were conducted per animal per sampling date to capture low parasitemia levels. The sensitivity of our nested PCR was determined to be 10^3^ infected erythrocytes per ml of blood, which corresponded to a parasitemia as low as 0.000016% [23].

To detect the presence of antibodies against *B*. *bovis* in nilgai and control calf serum, a recombinant protein representing the C-terminal region of the *B*. *bovis* rhoptry associated protein 1 (rRAP-1ct) was used as antigen in an indirect enzyme-linked immunosorbent assay as previously described [29]. The rRAP-1ct (100 µg) was diluted in 0.05 M carbonate-bicarbonate buffer, pH 9.6, and used to coat Immulon^™^ 2HB microtiter plates (Thermo Fisher Scientific) overnight at 4 °C. The plates were washed three times using 200 µl blocking buffer [0.2% I-Block^™^ (Thermo Fisher Scientific) in PBS with 0.1% Tween 20] and then blocked with 300 µl of the same buffer for 1 h at 30 °C. Nilgai serum samples were diluted 1/10 in blocking buffer and incubated in duplicate wells for 1 h at 30 °C. After incubation, the plates were washed five times in 200 µl blocking buffer, and 50 µl of a 1/1000 dilution of horseradish peroxidase-conjugated protein G (Invitrogen, Waltham, MS) was added to each well and incubated for 45 min at 30 °C. Plates were again washed four times using 200 µl blocking buffer and two times with 200 µl PBS with 0.1% Tween 20. SureBlue^™^ TMB (55 µl; SeraCare, Milford, MA) was added to each well, and the reactions were stopped after 10 min by adding 55 µl TMB stop solution (SeraCare). Absorbance was measured at 450 nm on a SpectraMax^®^ 190 plate reader (Molecular Devices, San Jose, CA), and average absorbance values with standard deviations were calculated and plotted. Pre- and postimmune *B*. *bovis*-infected serum from the positive control calf were used as a positive control in the assay. We assessed the antibody response at the following timepoints: (1) *B*. *bovis* stabilate: 0, 2, 4, 6, 8, 10, 14, and 21 dpi and (2) *B*. *bovis* larval sporozoite: 0, 2, 4, 6, 9, 12, 15, and 21 dpi, except for N011 who was sampled at 0, 2, 4, 6, and 15 dpi. The indirect rRAP-1-ELISA cut-off value was calculated as the mean plus two standard deviations of the nilgai pre-inoculate samples; optical density (OD) values above the cut-off were considered positive.

### Sub-inoculation of blood from *Babesia bovis*-challenged nilgai into naïve bovine calves

Before the endpoint of the experiment, whole blood (250 ml) from each nilgai calf was collected in acid citrate dextrose (ACD) blood collection bags and shipped overnight to the Animal Disease Research Unit (USDA-ARS, Pullman, WA). Blood from nilgai calves challenged with either the *B*. *bovis* blood stabilate or the *B*. *bovis* larval sporozoite were combined and inoculated intravenously into four spleen-intact calves. Each calf received 100 ml of blood containing 50 ml blood from each of two nilgai. Specifically, recipient beef calf C1809 was subinoculated with blood from N002 and N005, C1810 received blood from N006 and N008, C1862 received blood from N009 and N011, and C1863 received blood from N010 and N012. Recipient calves were monitored for 45 days for signs of bovine babesiosis, and blood was collected twice per week during this period. PCR was performed weekly to detect *B*. *bovis* infection as described above.

### In vitro *Babesia bovis* merozoite culture

To determine the ability of *B*. *bovis* parasites to expand in nilgai red blood cells (RBC), an in vitro culture growth curve was performed. Normal RBCs were isolated from peripheral blood of four nilgai prior to *B*. *bovis* challenge. The merozoite culture was initiated in a 48-well culture plate by introducing 10 µl of 5% *B*. *bovis*-infected bovine RBCs into 455 µl 40% bovine serum/HL1 (Lonza, Germany) culture medium with 10% PCV from the four nilgai RBC sources (N002, N005, N006, and N008). Culture medium supplemented with 10% PCV normal bovine RBCs was used as a positive control. Parasites were maintained in long-term microaerophilous stationary phase culture as previously described [30]. Percent of parasitized erythrocytes (PPE) was calculated every 24 h by blood smear and light microscopy to establish growth rates over 8 days.

### Histological examination of nilgai cerebral tissue

Nilgai calves were euthanized, and a gross necropsy was performed. Tissue samples of the brain, liver, and spleen were collected for histological examination. Formalin-fixed brain tissues from nilgai were paraffin embedded, cut into 5 µm sections, placed on glass slides, and stained with Giemsa (Washington Animal Disease Diagnostic Laboratory, Pullman, WA). Sections were evaluated to determine sequestration of *B*. *bovis*-infected RBCs in tissue capillaries.

## Results

**Fig. 1:**
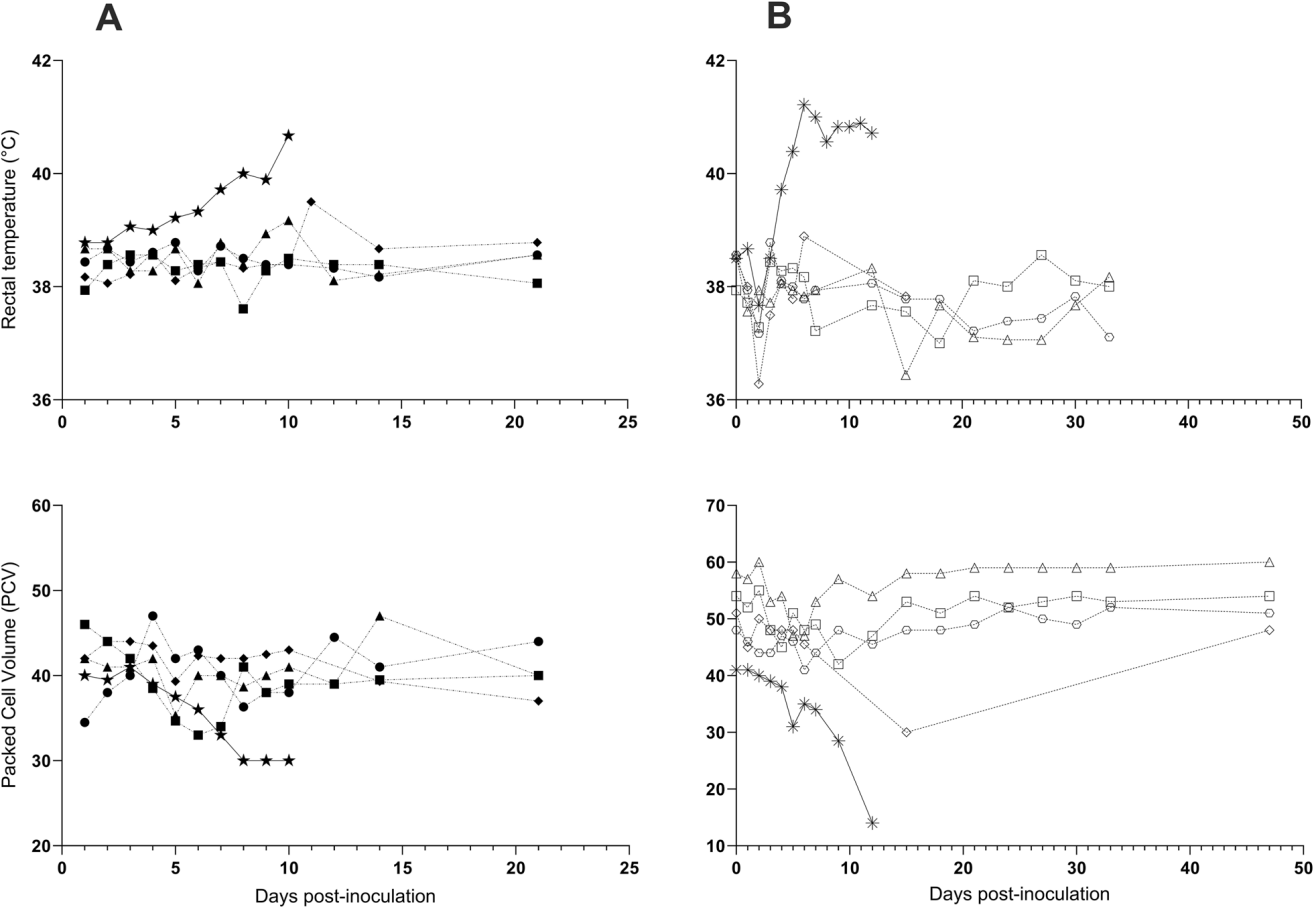
Rectal temperature (top panel) and packed cell volume (PCV; bottom panel) of nilgai antelope (*Boselephus tragocamelus*) and a positive control *Bos taurus* calf experimentally inoculated with a *Babesia bovis* blood stabilate (**A**) or a larval preparation containing *B*. *bovis* sporozoites (**B**). N002 (■), N005 (▲), N006 (◆), N008 (●), and positive control calf, C21 (★) and N009 (□), N010 (△), N011 (◊), N012 (○), and positive control calf, B11 (✳)

**Fig. 2:**
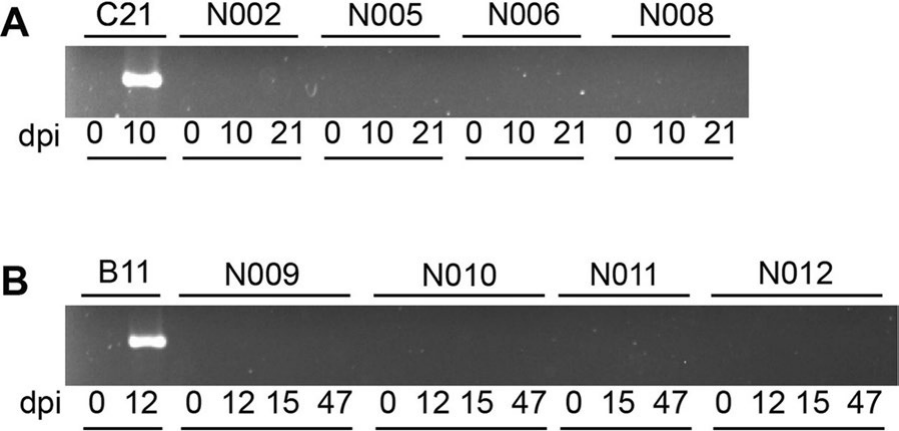
Assay to detect *Babesia bovis* by PCR targeting parasite 18S rRNA in nilgai antelope (*Boselephus tragocamelus*) challenged with either a *B*. *bovis* blood stabilate (**A**) or a larval preparation containing *B*. *bovis* sporozoites (**B**). Lanes C21 and B11 represent the positive-control *Bos taurus* calf used in each experiment. Representative results are depicted from the start of the study (0 dpi), the day on which the positive-control calf was euthanized (10 or 12 dpi), and the day on which the nilgai were euthanized (21 or 47 dpi). In (**B**), representative results include 15 dpi, as N011 could not be sampled at 12 dpi (see text). *dpi* day postinoculation

Upon challenge of nilgai (*n* = 4) with the *B*. *bovis* blood stabilate, no evidence of clinical infection was observed over the 21-day study period; body temperature and PCV remained normal and stable (Fig. 1). Elevated body temperature and decreasing PCV was observed in the positive control beef calf beginning 3 dpi, and the animal was euthanized at 10 dpi when body temperature exceeded 40.5 °C and PCV ≤ 30% (Fig. 1). DNA extracted from nilgai whole blood collected at multiple timepoints (1–12, 14, and 21 dpi) were PCR-negative (Figs. 2, S1). In contrast, *B*. *bovis* was detectable by PCR in the beef calf as early as 5 dpi (1 of 10) and confirmed at 7–10 dpi (10 of 10; Fig. S1).

Similarly, upon challenge of nilgai (*n* = 4) with the tick stage of *B*. *bovis*, no evidence of clinical infection was observed over the 47-day study period; body temperature and PCV remained normal and stable (Fig. 1). Elevated body temperature and decreasing PCV was observed in the positive control beef calf beginning 4 dpi, and the animal was euthanized at 12 dpi when body temperature was ≥ 40.5 °C and PCV ≤ 30% (Fig. 1). DNAs extracted from nilgai whole blood collected at multiple timepoints (1–7, 12, 15, 18, 21, 24, 27, 30, 33, and 47 dpi) were PCR-negative (Figs. 2, S2). In contrast, *B*. *bovis* was detectable by PCR in the beef calf as early as 6 dpi (2 of 10) and confirmed at 7, 9, and 12 dpi (10 of 10; Figs. 2, S2).

**Fig. 3:**
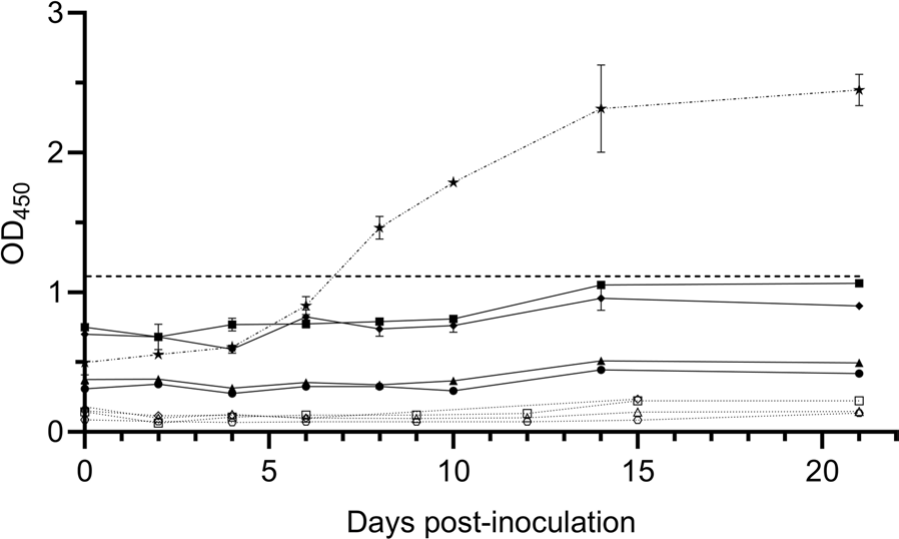
Detection of anti-*Babesia bovis* antibodies by indirect ELISA. Sera from nilgai antelope (*Boselephus tragocamelus*) challenged with a *B*. *bovis* blood stabilate (N002, N005, N006, and N008) or a larval preparation containing *B*. *bovis* sporozoites (N009, N010, N011, andN012) were evaluated by indirect ELISA using a *B*. *bovis* recombinant protein (rRAP1-ct) as antigen. The mean optical density (OD) values ± SD are presented as a function of days postinoculation for each animal. The cut-off value (---) for a positive test was calculated as the mean plus two standard deviations of the nilgai pre-inoculate samples. C1703: archived positive control sera from a *Bos taurus* calf that survived infection with *B*. *bovis* (not this study). N002 (■), N005 (▲), N006 (◆), and N008 (●); N009 (□), N010 (△), N011 (◊), and N012 (○); and positive control calf, C1703 (★)

Sera collected over the course of the 21 day (blood stabilate) or 47 day (tick stage) experiments were analyzed by an indirect ELISA on the basis of *B*. *bovis* RAP-1 to detect production of antibodies in response to *B*. *bovis*. Indirect ELISAs indicated that anti-RAP-1 antibodies were not detectable in nilgai sera at these timepoints but were detected in serum from a positive-control beef calf (Fig. 3).

**Fig. 4:**
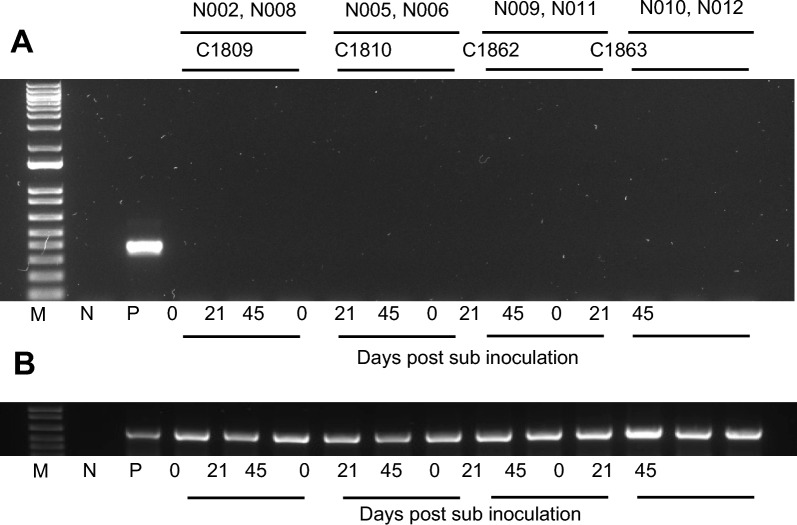
PCR analysis of susceptible *Bos taurus* calves sub-inoculated with blood from nilgai antelope (*Boselephus tragocamelus*) challenged with *Babesia bovis*. Nilgai: N002, N005, N006, and N008 were challenged with *B*. *bovis* blood stabilate, and N009, N010, N011, and N012 were challenged with a larval preparation containing *B*. *bovis* sporozoites. Recipient calves C1809, C1810, C1862, and C1863 were sub-inoculated with blood from *B*. *bovis*-challenged nilgai (see Methods). Amplicons were resolved on 2% agarose. PCR targeting (**A**) 18S ribosomal RNA and (**B**) bovid cytochrome b gene. M: 1 kb plus DNA ladder maker, *N* negative control, *P* positive control

**Fig. 5:**
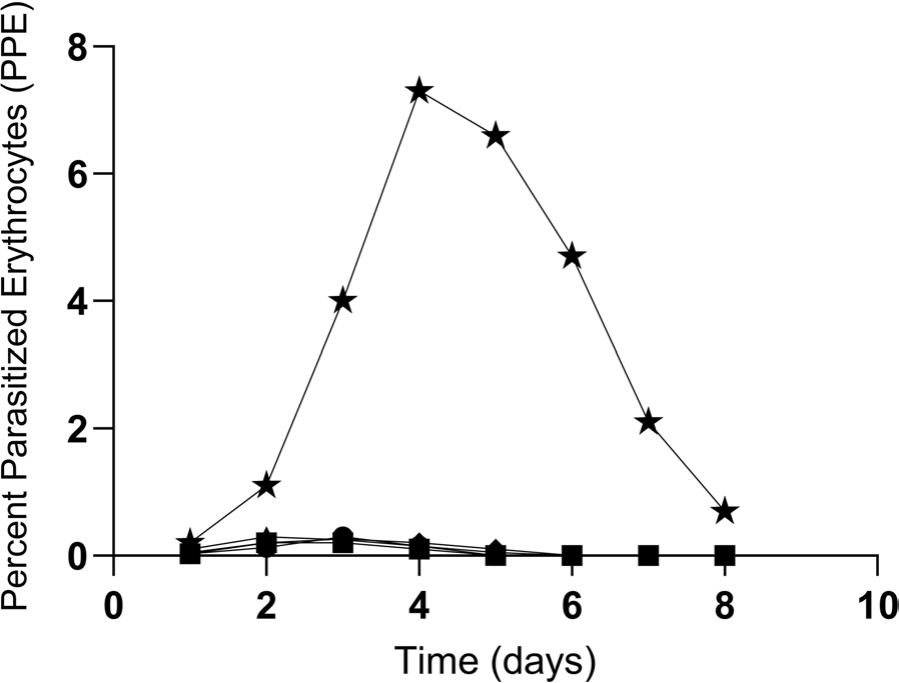
In vitro culture of *Babesia bovis* merozoites with host red blood cells (RBCs). The culture was initiated using bovine serum-HL1 culture medium with 10% PCV of nilgai antelope (*Boselephus tragocamelus*) or cattle (*Bos taurus*) RBCs. Culture inoculated with *B*. *bovis*-infected *Bos* RBCs was used as a positive control. Nilgai and *B*. *taurus* RBCs were from animals that had not been challenged with *B*. *bovis*. Percent of parasitized erythrocytes (PPE) was calculated daily by blood smear and light microscopy to establish growth rates over 8 days. N002 (■), N005 (▲), N006 (◆), N008 (●) and positive control calf C-1768 (★)

To rule out the possibility that parasites were present at a level undetectable by PCR, blood from nilgai challenged with either *B*. *bovis* merozoites or the tick stage of the parasite were inoculated into spleen-intact calves. Clinical signs of infection were not observed, and PCR results were negative in the recipient cattle throughout the experiment (Fig. 4). Further, using an in vitro culture approach, nilgai RBCs were not colonized/infected by *B*. *bovis* merozoites. In contrast, the control bovine RBCs were colonized with a peak of 7.3% observed at day 4 (Fig. 5).

**Fig. 6:**
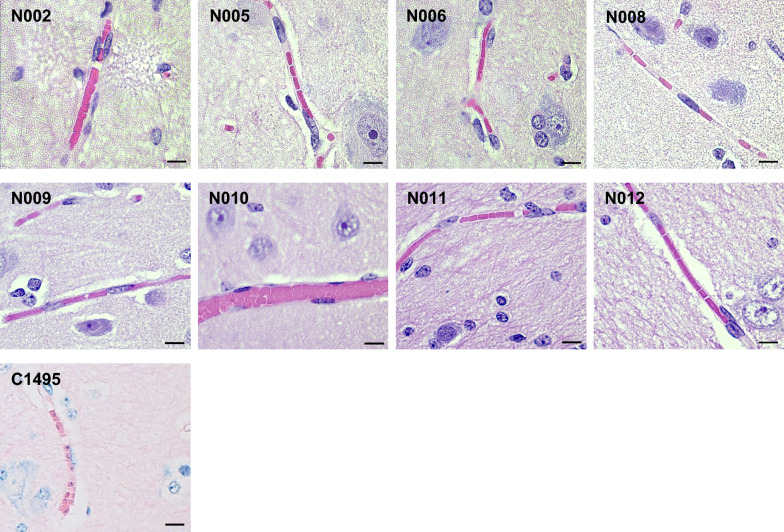
Histological examination of cerebrum tissue from four nilgai antelope (*Boselephus tragocamelus*) and a positive control cattle (*Bos taurus*). *Babesia bovis* parasites were visualized as small circular objects within red blood cells in the capillaries of the cerebrum, indicating infection in the positive control animal. No such infection was observed in the cerebrum tissue sections of nilgai. Arrows indicates *B. bovis-*infected erythrocytes. Scale bar: 10 µm

*Babesia bovis*-infected erythrocytes sequester in the brain capillaries and as a result are a likely organ in which to identify the parasite [31]. Histological evaluation of the brain from nilgai calves challenged with either the blood stabilate or tick stage of *B*. *bovis* did not show any evidence of infection (Fig. 6).

## Discussion

Protecting the US cattle herd from livestock diseases is essential to maintaining a sustainable beef industry. Bovine babesiosis is a tickborne disease that can cause mortality rates of up to 90% [32], which would be likely given that US cattle are immunologically naïve to *Babesia*. A reemergence of bovine babesiosis would be economically devastating to the industry, as cattle are ranked sixth in agricultural commodities produced in the USA and the USA maintains the largest fed-cattle industry in the world, with a value of $78 billion in farm gate cash receipts in 2022 [33]. The risk is further emphasized by evidence that stray Mexico-origin cattle intercepted in the Texas–Mexico transboundary region are infected with *B*. *bovis* or *B*. *bigemina* [34]. Additionally, nilgai antelope populations are highest in the southeasternmost counties of Texas (Cameron and Willacy counties), and they are routinely found to be infested with *R*. (*B*). *microplus* in this area [11, 12] as well as in the Tick Eradication Quarantine Area that is an integral region within the CFTEP [5, 15]. The documented long-range movement of nilgai antelope [13, 35] increases the possibility that these nilgai could transport infected ticks originating from Mexico, where *B*. *bovis* and *B*. *bigemina* are endemic. Given the overlapping habitat of alternative hosts with primary cattle hosts, it is imperative to understand the impacts of exotic species that are a new addition to the ecology and epidemiology of this disease system.

Aside from cattle within the *Bos* genus, yak (*Bos grunniens*) are susceptible to *B*. *bovis* infection, serving as reservoirs for the parasite [36]. There is evidence of other ungulates in the Family Bovinae, related to *Bos* spp. bovines, that are also susceptible to infection by *Babesia* spp., including bushbuck (*Tragelaphus scriptus*) from which characteristic intraerythrocytic protozoa were identified [37]. The susceptibility of water buffaloes (Bovinae: *Bubalus bubalis*) to infection by *B*. *bovis* was demonstrated by experimental inoculation with 10^8^ infected erythrocytes [38], which is comparable to that used in our current study. The inoculated, spleen-intact water buffalo displayed no clinical signs of infection, and the parasite was only detectable in a subset of animals using either PCR or indirect ELISA [38]. This is supported by field observations in which *B*. *bovis*-infected water buffalo raised together with infected cattle were mildly symptomatic or asymptomatic, had lower percentage of animals infected relative to cattle, and exhibited no clinical signs of infection [39, 40]. Interestingly, splenectomized water buffalo experimentally inoculated with *B*. *bovis* reportedly displayed elevated temperature and reduced hematocrit that coincided with increased percent parasitized erythrocytes [41], suggesting there may be a heightened, antiparasitic response facilitated by the immune system in this species.

Microscopic analyses of thin blood smears from a nilgai in India identified intraerythrocytic protozoa characteristic of *Babesia* spp. [42], but molecular confirmation of the species was not reported and it could represent an as-yet unidentified host-specific species. While PCR-positive nilgai were detected in northern Mexico [20], sequence confirmation was not available for the amplicons. In contrast, a molecular diagnostic survey of 200 nilgai in the Texas–Mexico transboundary region bordering the northeastern state of Tamaulipas, Mexico, did not identify PCR-positive hosts [12].

In the current study, we challenged nilgai with either *B*. *bovis* merozoites or a sporozoite preparation, and the nilgai did not exhibit clinical signs of infection. Further, *B*. *bovis* was not detected by PCR and antibodies to the parasite did not develop in nilgai, irrespective of the stage used in the challenge. Additional results supported these observations, including the inability of a *B*. *bovis* merozoite culture to colonize nilgai RBCs, the inability to recover *Babesia* from nilgai by sub-inoculation into susceptible bovine calves, and the absence of detectable *B*. *bovis* parasites within RBCs in the capillaries of the brain. While in vitro culture studies described colonization of white-tailed deer RBCs by *B*. *bovis*, this result was critically dependent on culture supplementation with bovine serum [43]. This suggested that in vivo infection of white-tailed deer was unlikely, which was validated by experimental infection studies [23, 44]. Here, a similar in vitro culture approach indicated that nilgai RBCs were not colonized by *B*. *bovis* even when supplemented with bovine serum, further supporting the experimental inoculation results. The host immune response can differ when pathogen is delivered by needle inoculation versus an arthropod bite; however, inclusion of cattle infected by needle inoculation supported the validity of the intravenous introduction. The challenge strain used in our study is known to infect cattle and cause disease with a minimum dose of 10 *B*. *bovis* infected red blood cells. We elected to infect the nilgai with a significantly higher dose than would infect cattle as a means of increasing the probability of infection, should nilgai be susceptible, and in keeping with experimental inoculations of other family Bovinae species [38, 40]. Collectively, the results indicate that nilgai are not susceptible to infection by *B*. *bovis*.

## Conclusions

Nilgai do not appear to be susceptible to infection with a strain of *B*. *bovis* that is lethal to cattle, indicating that it does not play a significant role in the epidemiology of bovine babesiosis. Complementary experimental inoculation studies to evaluate nilgai susceptibility to infection with *B*. *bigemina* are warranted, as this species of *Babesia* is transmitted by a different tick stage (nymph) and is endemic to Mexico. Further, inoculation studies of non-*Bos* spp. in the family Bovinae with *B*. *bigemina* indicated that water buffaloes, East African buffalo (*Syncerus caffer*), and American bison (*Bison bison*) are susceptible to infection. Ultimately, regardless of the role that exotic ungulates have in the transmission of bovine babesiosis, tick control on these alternative hosts remains a critical priority owing to their potential to disseminate ticks over long distances.

### Supplementary Information

Supplementary Material 1: Figure S1. Assay to detect *Babesia bovis* by PCR targeting parasite 18S rRNA in nilgai antelope (*Boselephus tragocamelus*) challenged with a *B*. *bovis* blood stabilate. Figure S2. Assay to detect *Babesia bovis* by PCR targeting parasite 18S rRNA in nilgai antelope (*Boselephus tragocamelus*) challenged with a larval preparation containing *B*. *bovis* sporozoites.

## Data Availability

All data used to generate conclusions and analyzed in this study are represented in figures and supplementary information.

## Abbreviations

CFTEP: Cattle Fever Tick Eradication Program
dpi: Days postinoculation
OD: Optical density
PCV: Packed cell volume
PPE: Percent parasitized erythrocytes
RBC: Red blood cell
USDA: US Department of Agriculture
