# Supplementary material for: Nilgai antelope display no signs of infection upon experimental challenge with a virulent Babesia bovis strain
Source: Parasit Vectors. 2024 Jun 1;17:245. doi: 10.1186/s13071-024-06316-3 (PMC11144341; doi:10.1186/s13071-024-06316-3)
Supplement: Supplementary file 1 — Supplementary Material 1: Figure S1. Assay to detect Babesia bovis by PCR targeting parasite 18S rRNA in nilgai antelope (Boselephus tragocamelus) challenged with a B. bovis blood stabilate. Figure S2. Assay to detect Babesia bovis by PCR targeting parasite 18S rRNA in nilgai antelope (Boselephus tragocamelus) challenged with a larval preparation containing B. bovis sporozoites. [file 13071_2024_6316_MOESM1_ESM.pdf]

<u>Figure S1.</u> Assay to detect *Babesia bovis* by PCR targeting parasite 18S rRNA in nilgai antelope (*Boselephus tragocamelus*) challenged with a *B. bovis* blood stabilate. Results from Nilgai 002, 005, 006, 008, and C-21 are presented, ten replicate reactions per animal per timepoint. C-21 is the positive control *Bos taurus* calf used during this experimental challenge. MW: molecular weight marker; + or + ctrl: positive control DNA from a *B. bovis*-infected calf W-233; NTC: no template control

Day 5

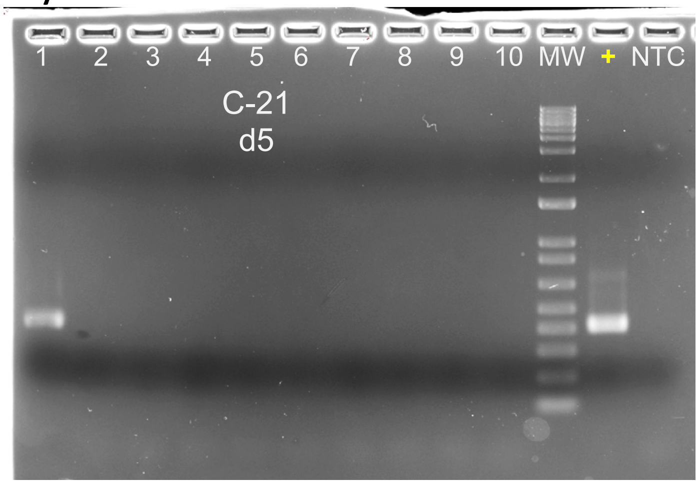

Day 6

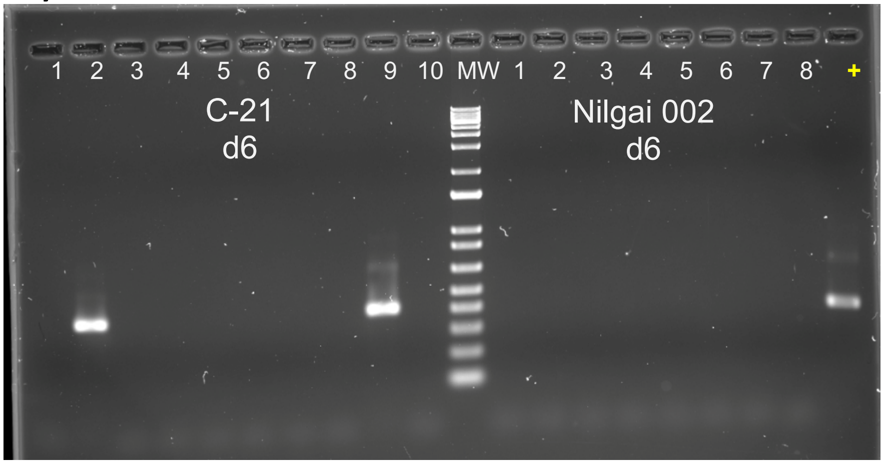

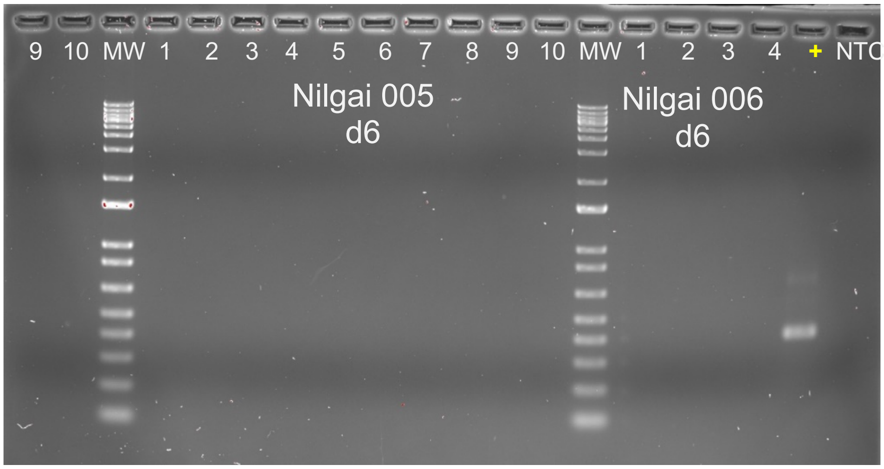
9 10 MW 1 2 3 4 5 6 7 8 9 10 MW 1 2 3 4 + NTC
Nilgai 005
d6
Nilgai 006
d6

Day 7 (C-21)

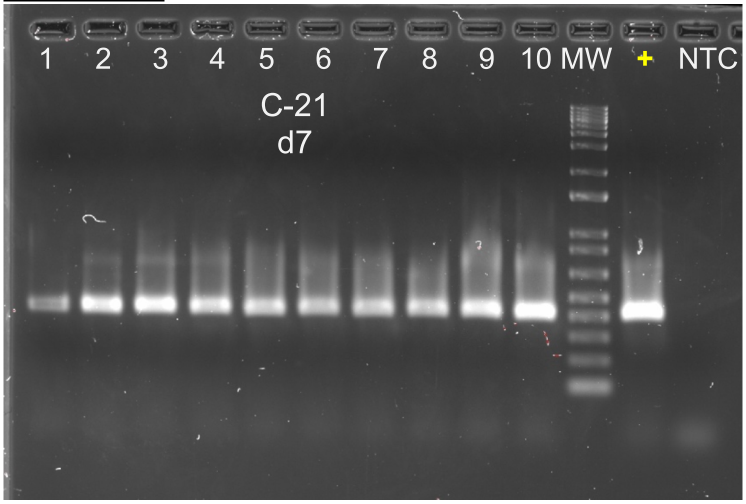

Day 8 (C-21)

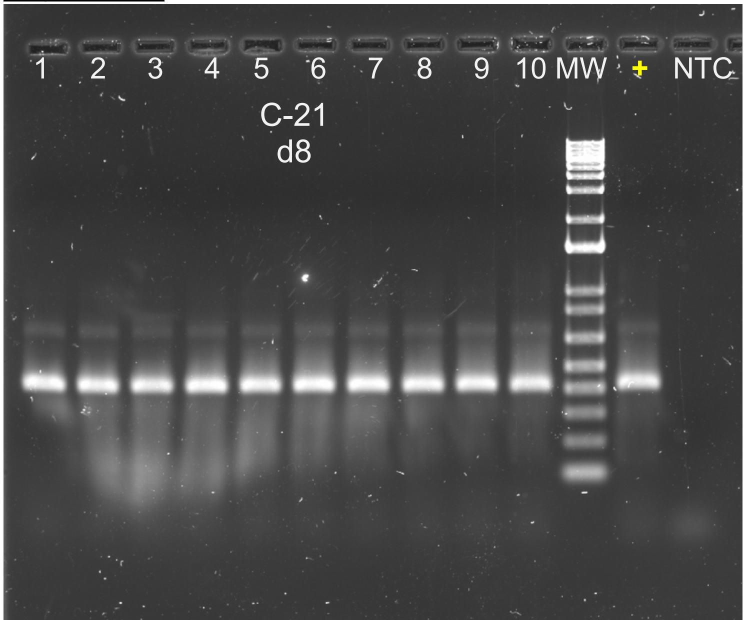

Day 7, Day 8 Nilgai

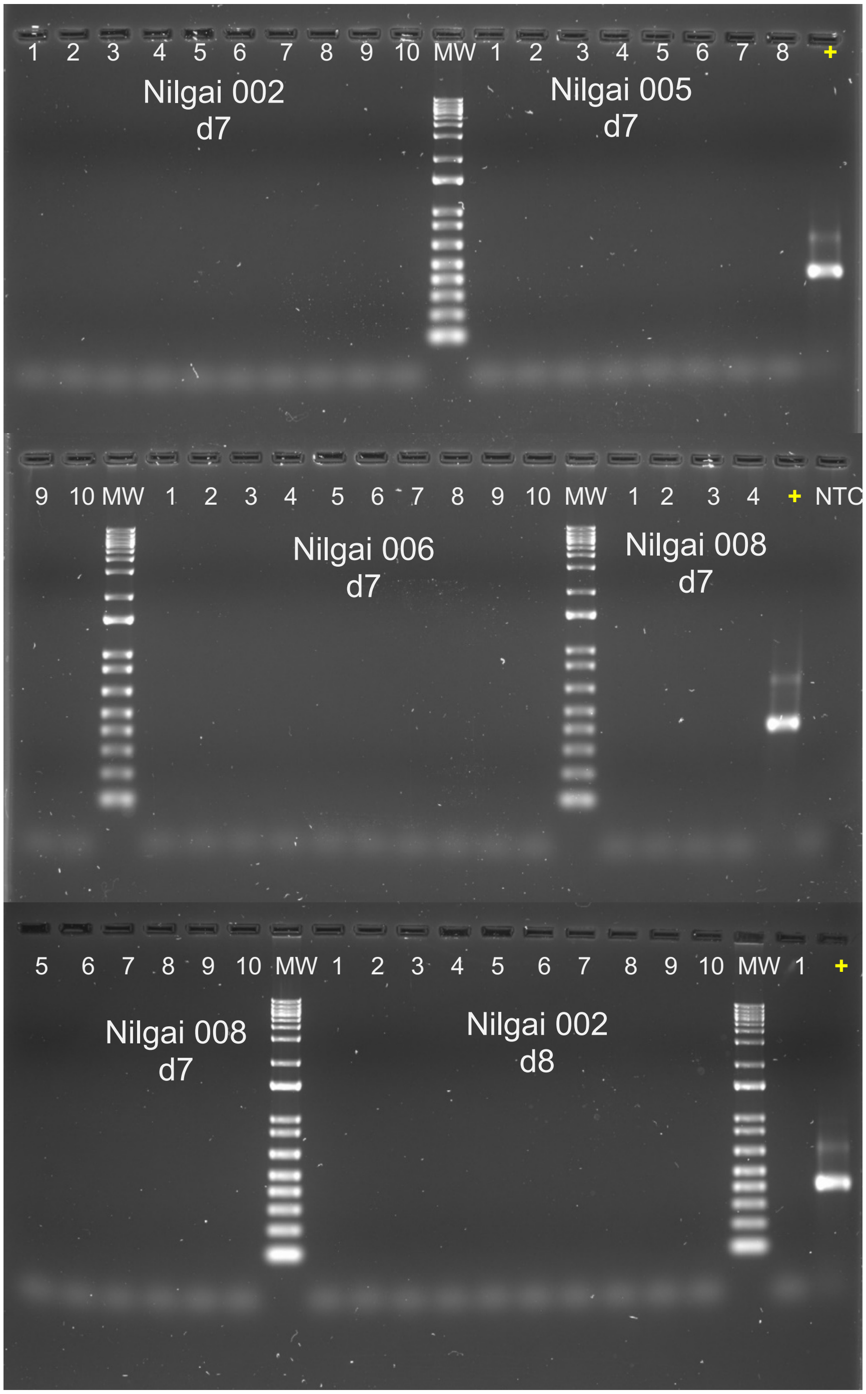

Day 7, Day 8 Nilgai (contd):

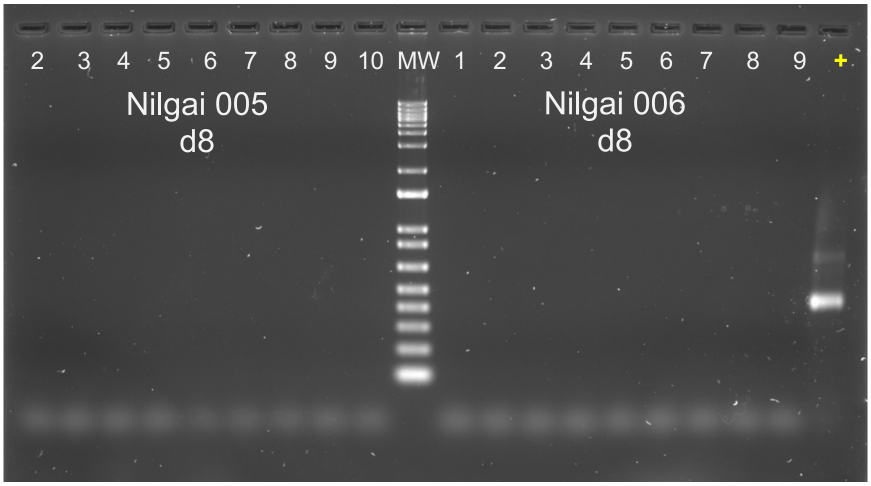

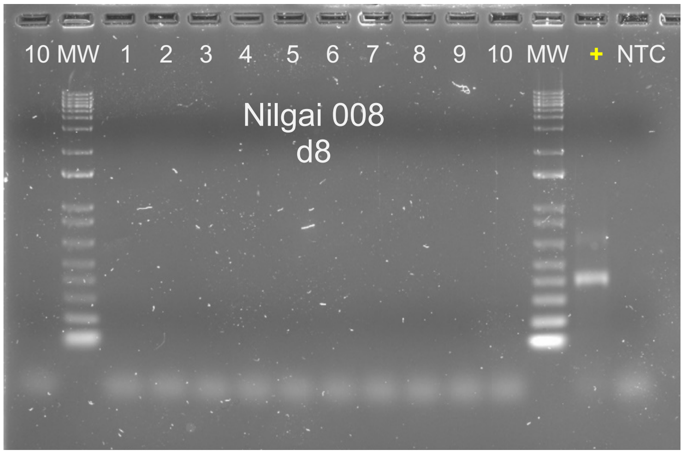

Day 9 (C-21)

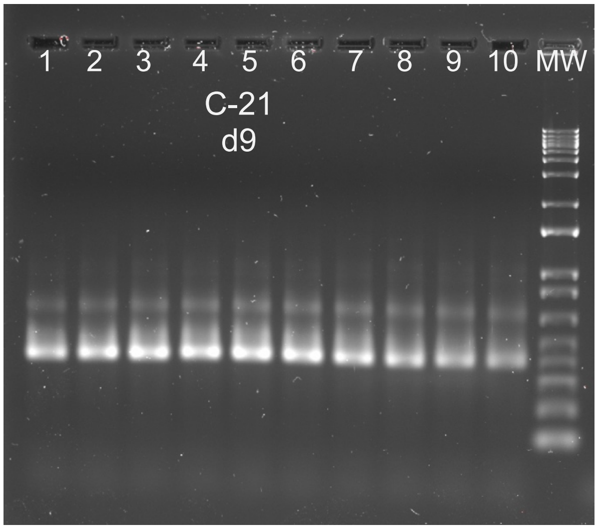

Day 10 (C-21)

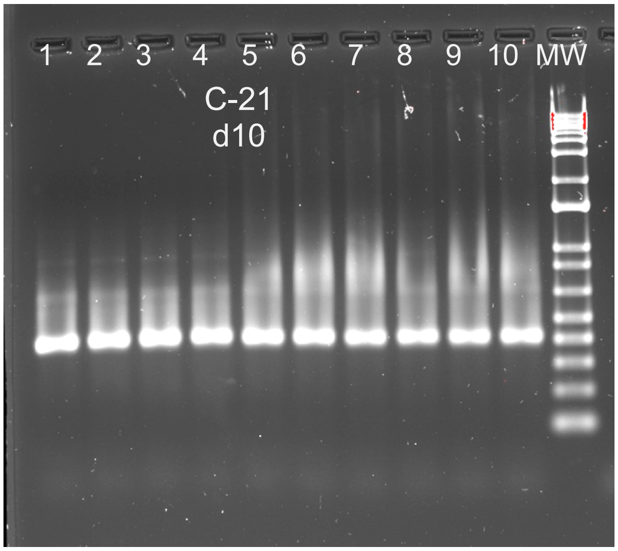

<u>**N002 (d9 – d21 dpi)**</u>

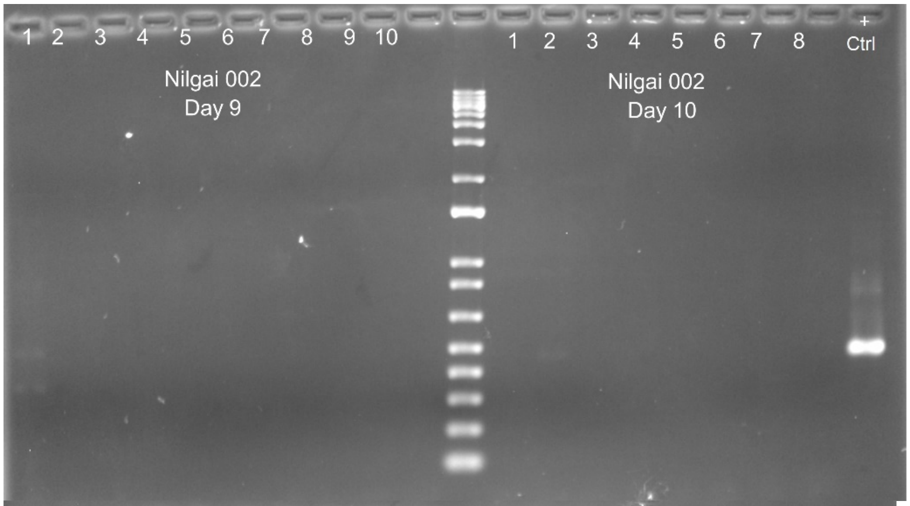

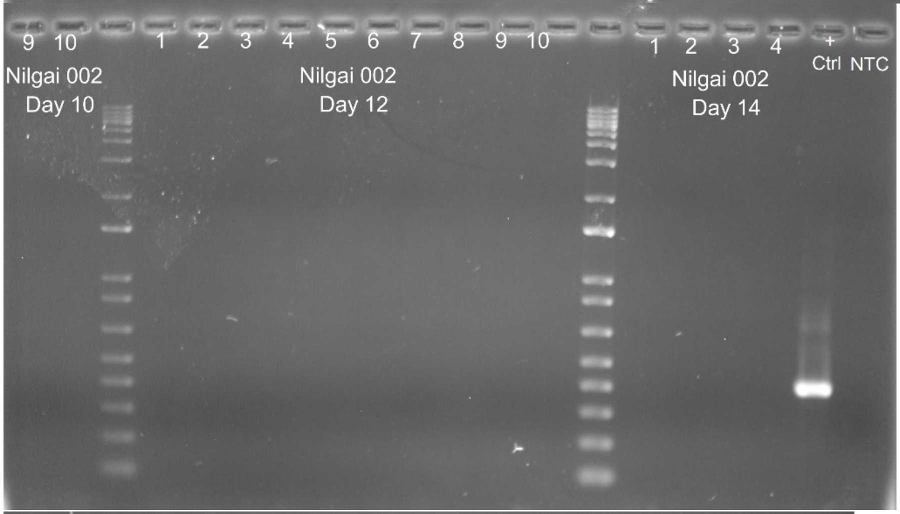

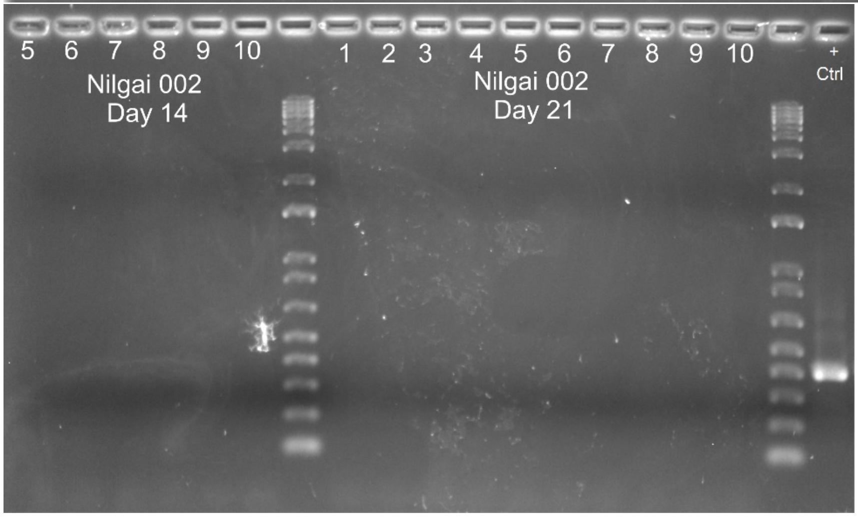

N005 (d9 – d21 dpi)

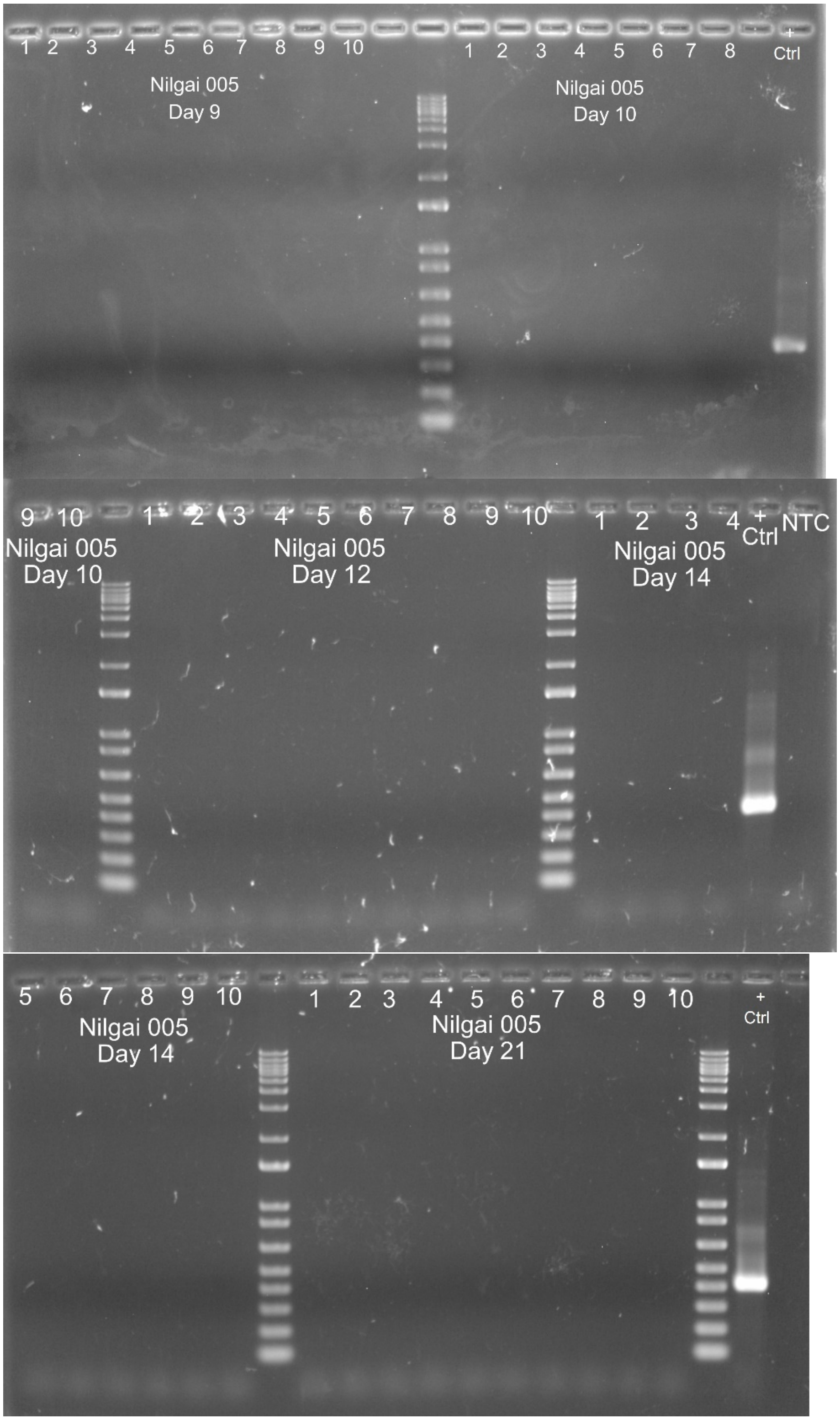

N006 (d9 – d21 dpi)

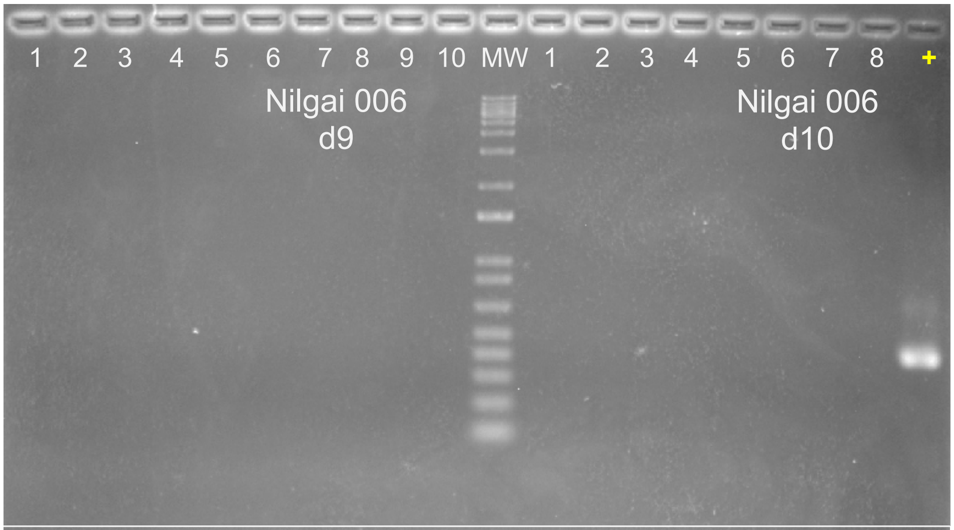

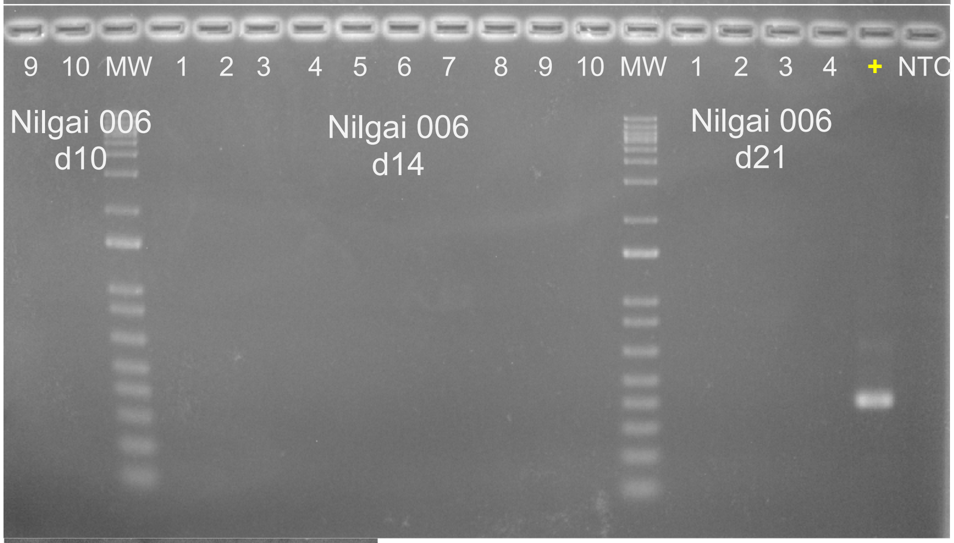

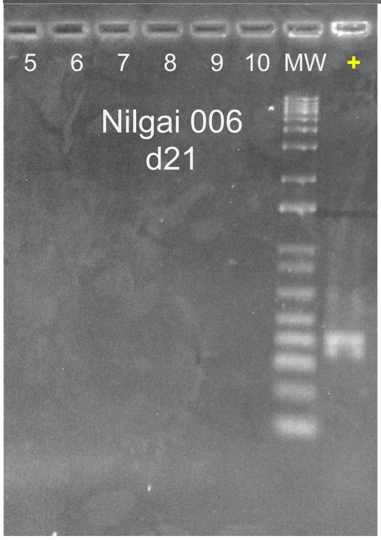

N008 (d9 – d21 dpi)

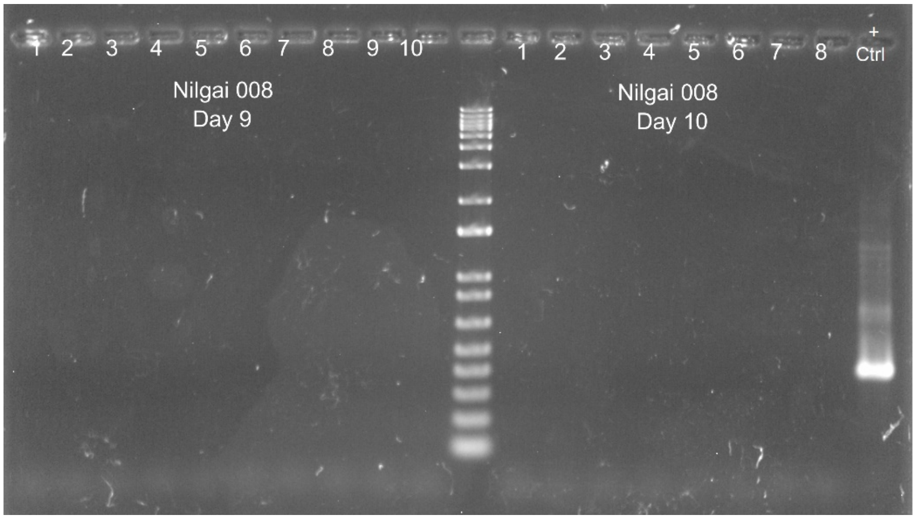

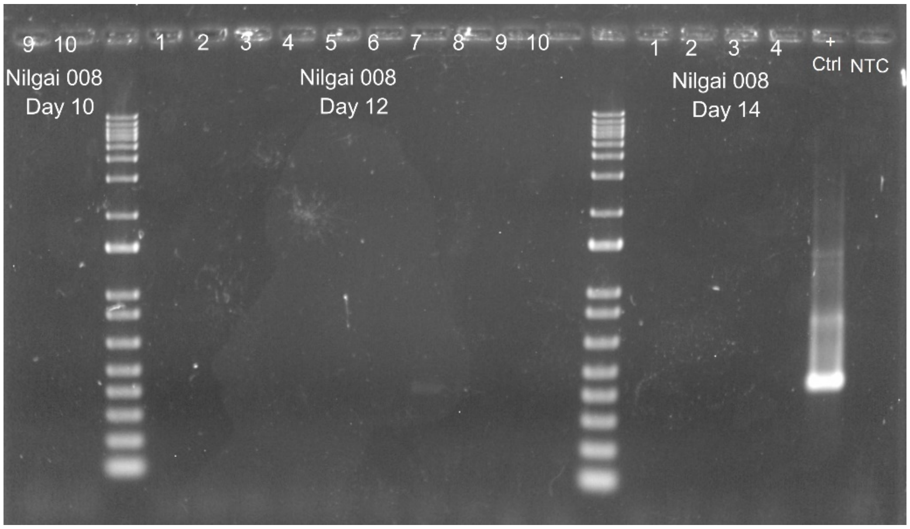

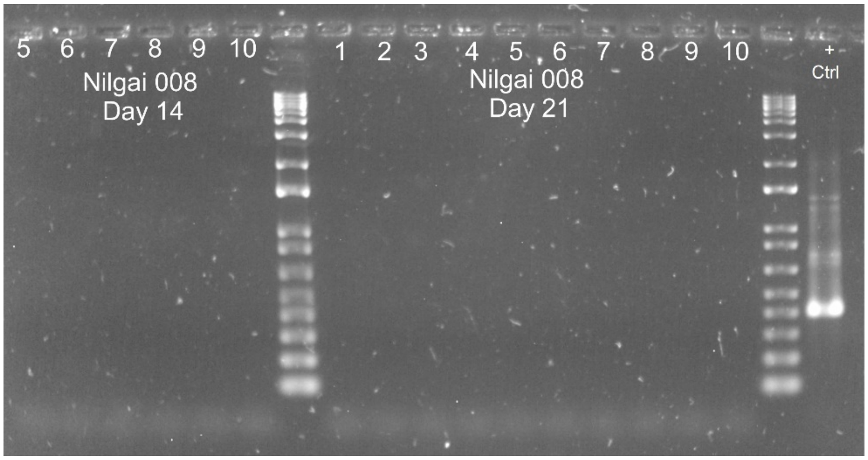

<u>Figure S2.</u> Assay to detect *Babesia bovis* by PCR targeting parasite 18S rRNA in nilgai antelope (*Boselephus tragocamelus*) challenged with a larval preparation containing *B. bovis* sporozoites. Results from Nilgai 009, 010, 011, 012, and B-11 are presented, ten replicate reactions per animal per timepoint. B-11 is the positive control *Bos taurus* calf used during this experimental challenge. + ctrl: positive control DNA from a *B. bovis*-infected calf W-233; NTC: no template control

<u>Day 6</u>

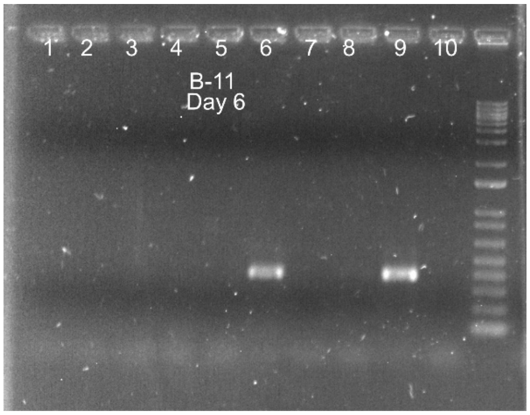

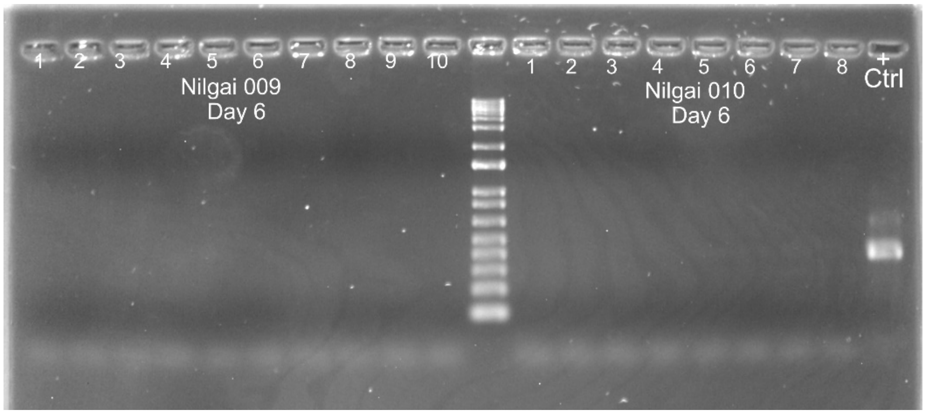

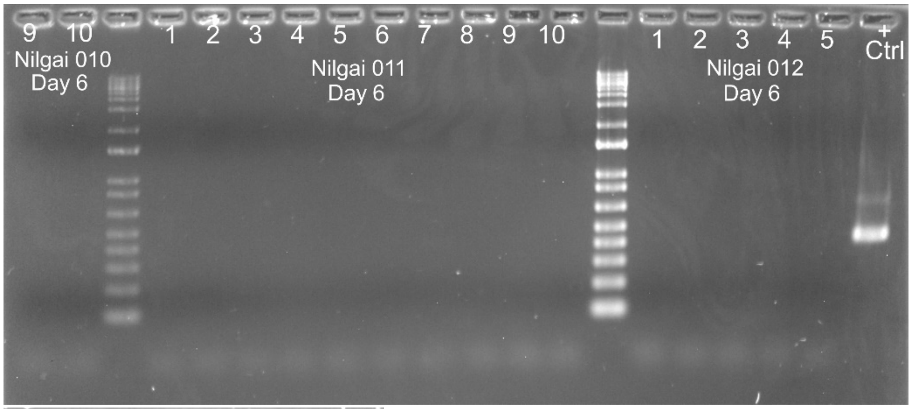

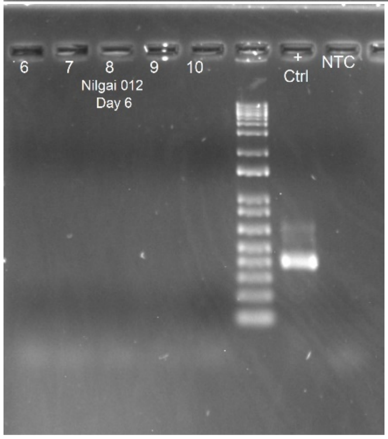

Day 7

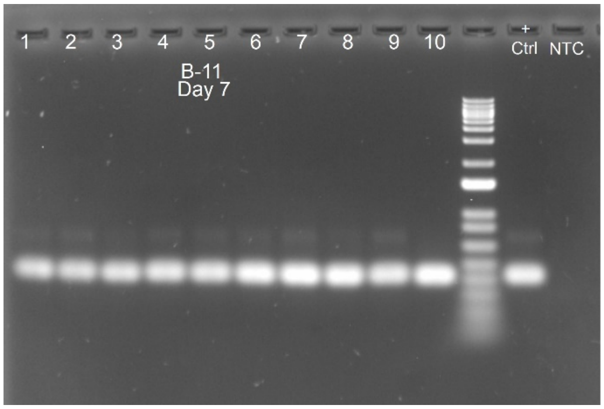
1 2 3 4 5 6 7 8 9 10
+ Ctrl
NTC
B-11
Day 7

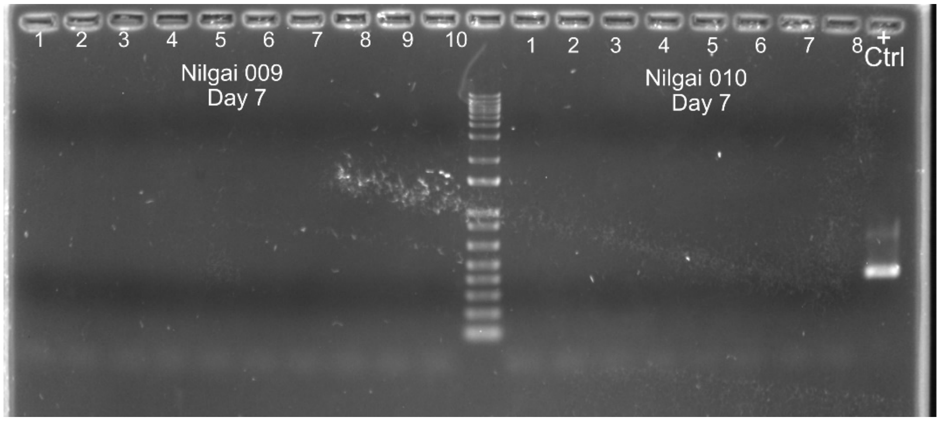
1 2 3 4 5 6 7 8 9 10
Nilgai 009
Day 7
1 2 3 4 5 6 7 8
+ Ctrl
Nilgai 010
Day 7

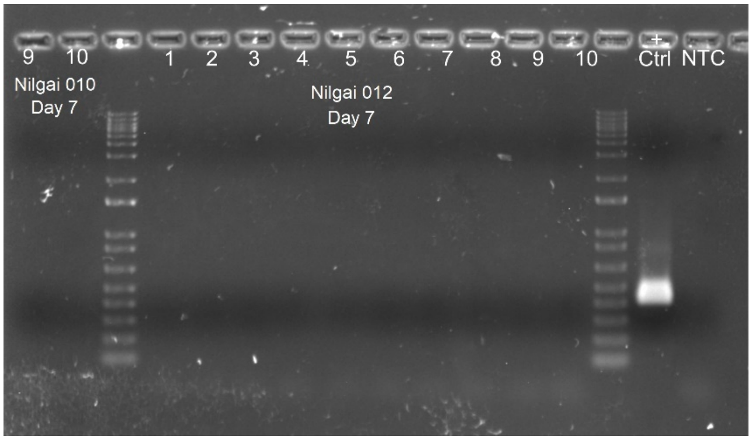
9 10
Nilgai 010
Day 7
1 2 3 4 5 6 7 8 9 10
Nilgai 012
Day 7
+ Ctrl
NTC

Day 9

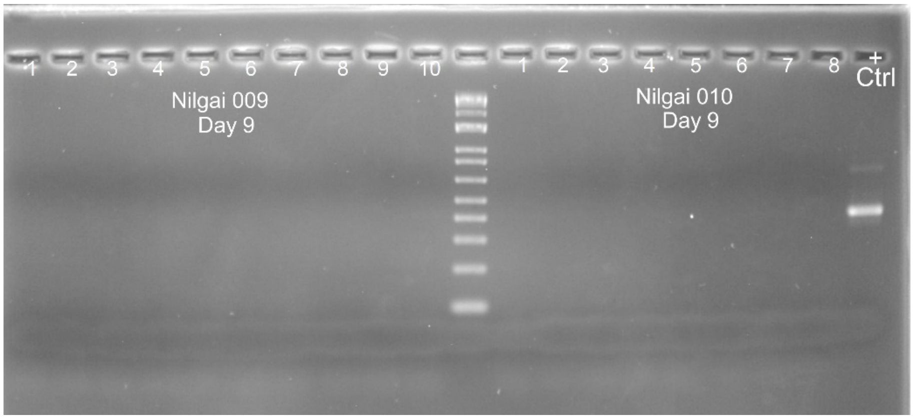
1 2 3 4 5 6 7 8 9 10
Nilgai 009
Day 9
1 2 3 4 5 6 7 8
Nilgai 010
Day 9
+
Ctrl

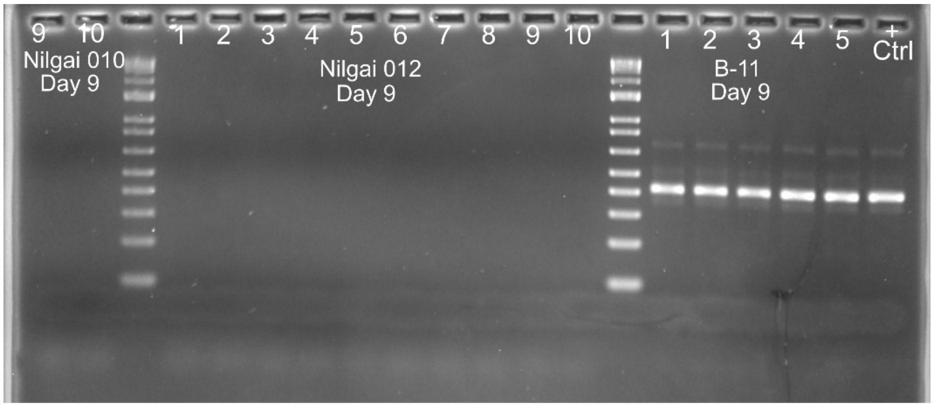
9 10
Nilgai 010
Day 9
1 2 3 4 5 6 7 8 9 10
Nilgai 012
Day 9
1 2 3 4 5
B-11
Day 9
+
Ctrl

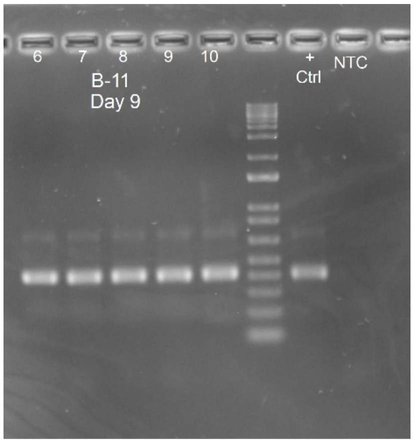
6 7 8 9 10
B-11
Day 9
+
Ctrl
NTC

Day 12

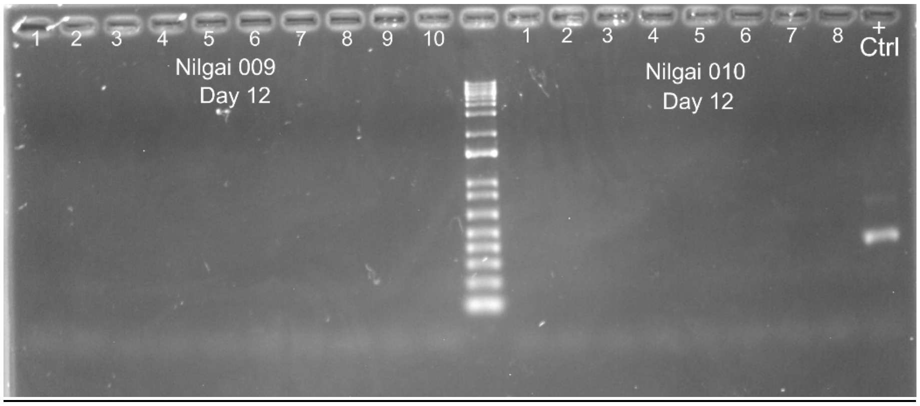
1 2 3 4 5 6 7 8 9 10
Nilgai 009
Day 12
1 2 3 4 5 6 7 8
+ Ctrl
Nilgai 010
Day 12

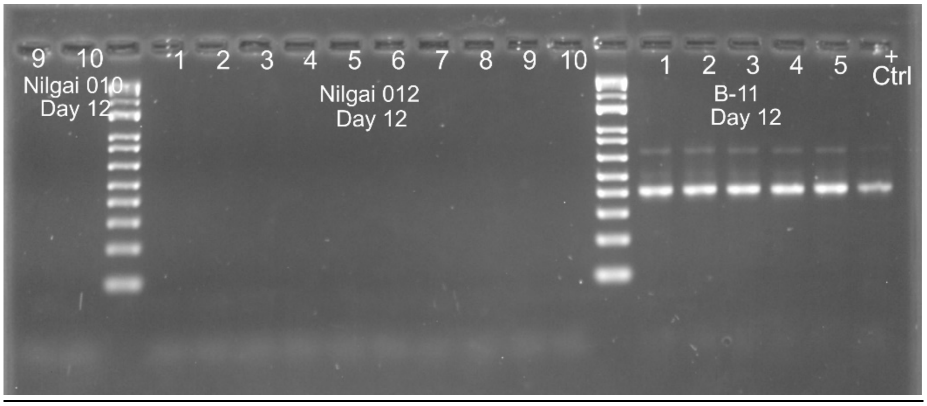
9 10
Nilgai 010
Day 12
1 2 3 4 5 6 7 8 9 10
Nilgai 012
Day 12
1 2 3 4 5
+ Ctrl
B-11
Day 12

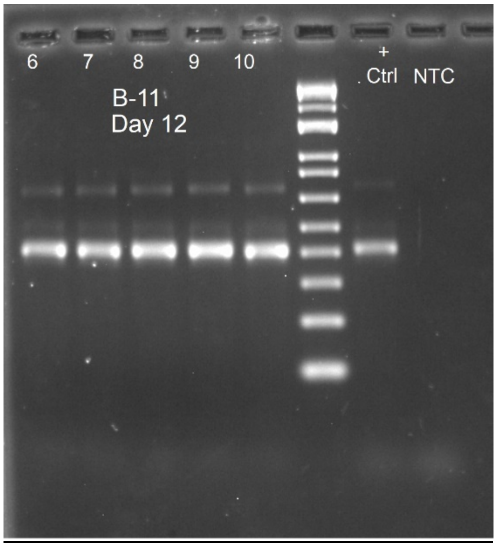
6 7 8 9 10
B-11
Day 12
+ Ctrl
NTC

N009 (d21 – d30 dpi)

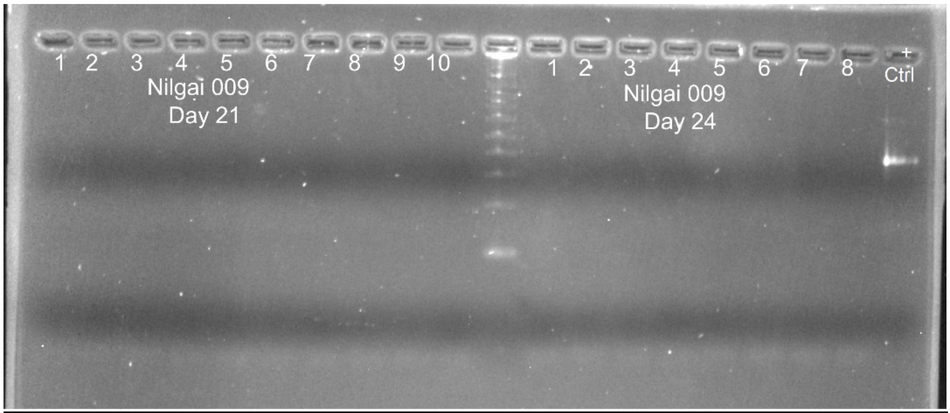

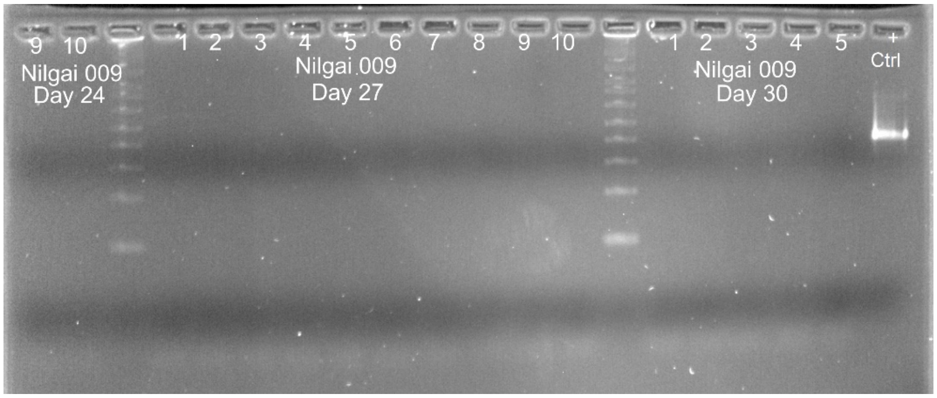

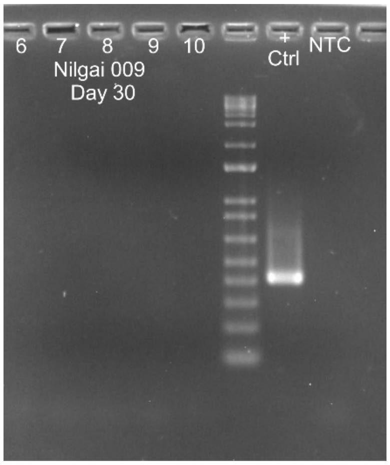

N010 (d21 – d30 dpi)

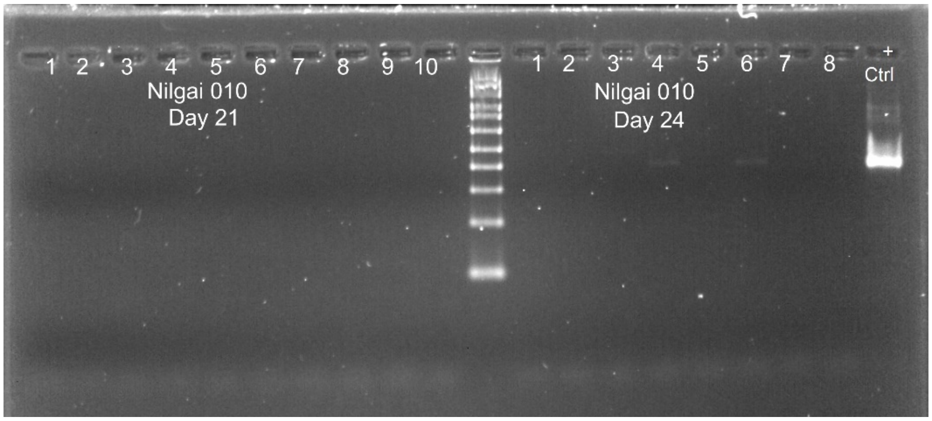

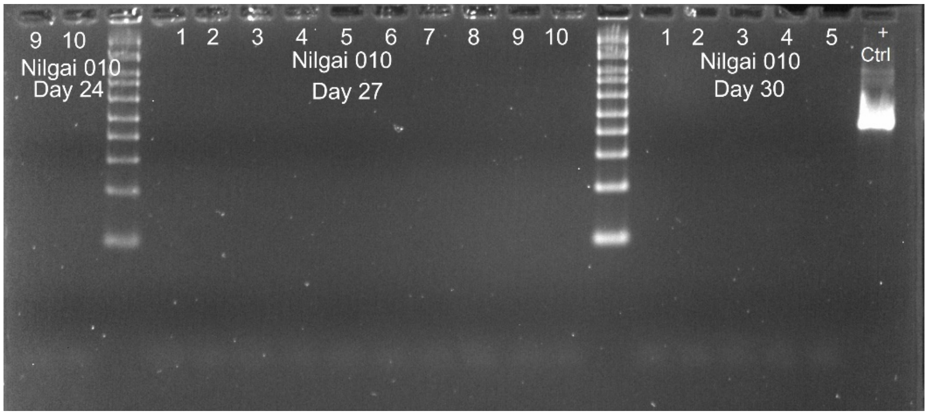

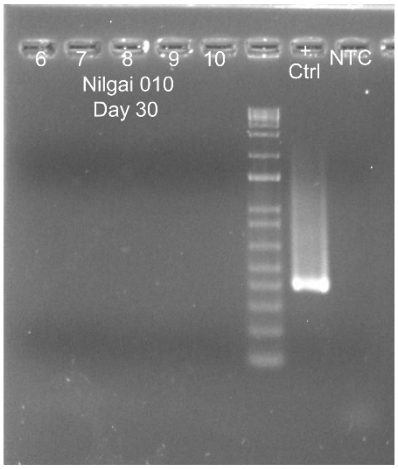

N012 (d21 – d30 dpi)

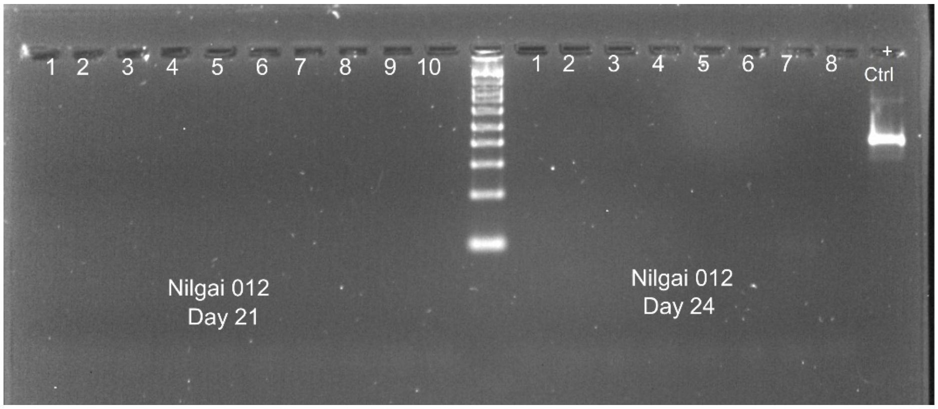

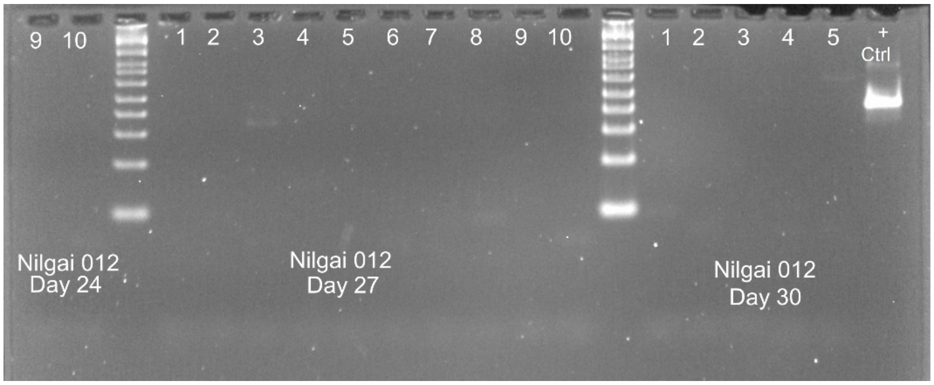

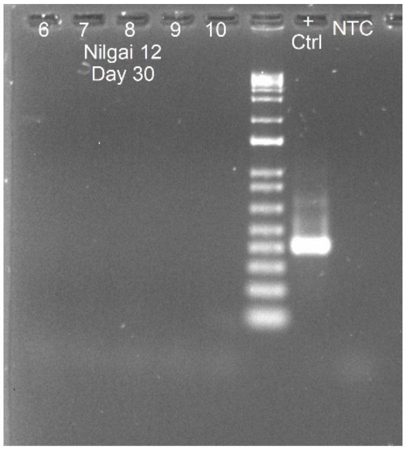

Day 33

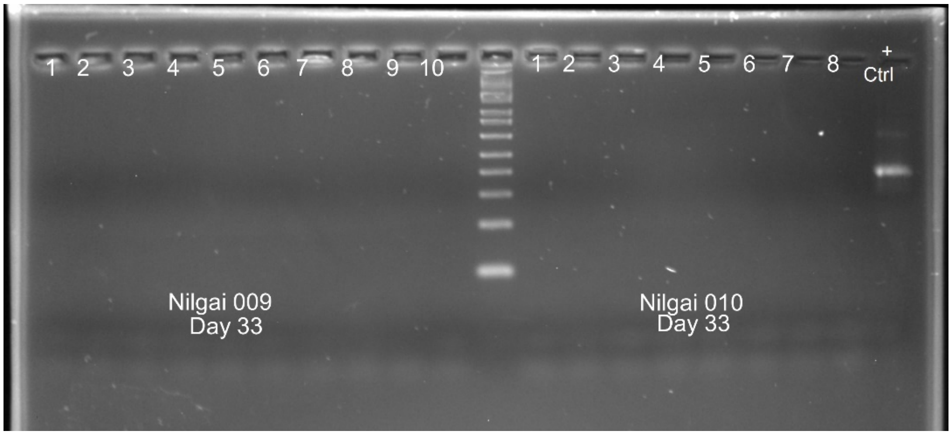
1 2 3 4 5 6 7 8 9 10
1 2 3 4 5 6 7 8
+ Ctrl
Nilgai 009
Day 33
Nilgai 010
Day 33

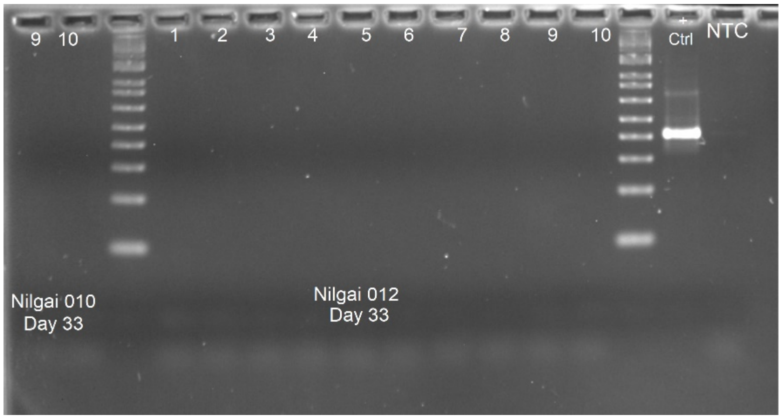
9 10
1 2 3 4 5 6 7 8 9 10
+ Ctrl
NTC
Nilgai 010
Day 33
Nilgai 012
Day 33

Day 47

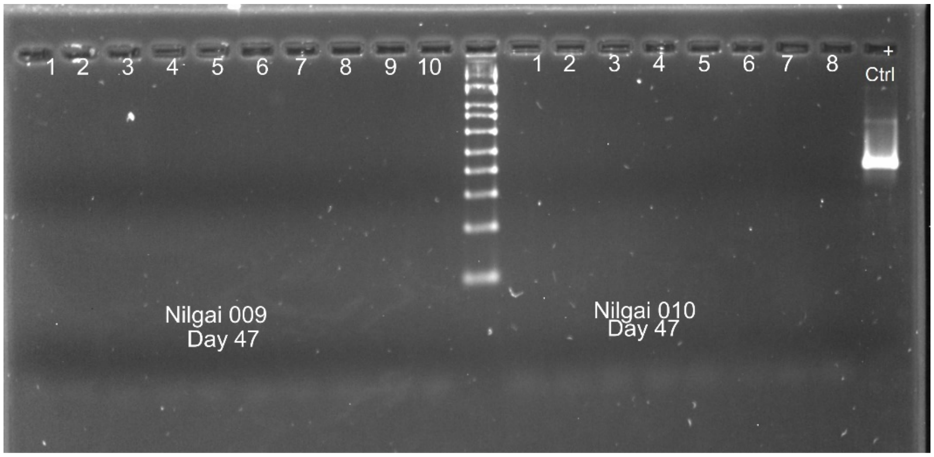
1 2 3 4 5 6 7 8 9 10
1 2 3 4 5 6 7 8
+
Ctrl
Nilgai 009
Day 47
Nilgai 010
Day 47

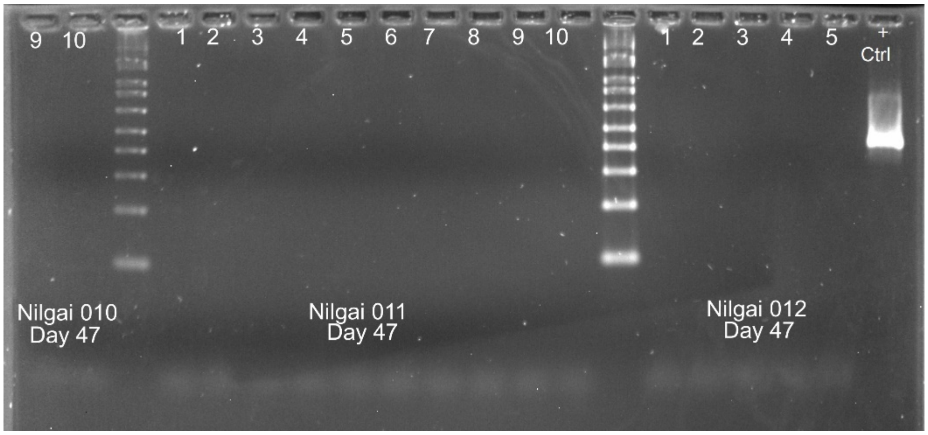
9 10
1 2 3 4 5 6 7 8 9 10
1 2 3 4 5
+
Ctrl
Nilgai 010
Day 47
Nilgai 011
Day 47
Nilgai 012
Day 47

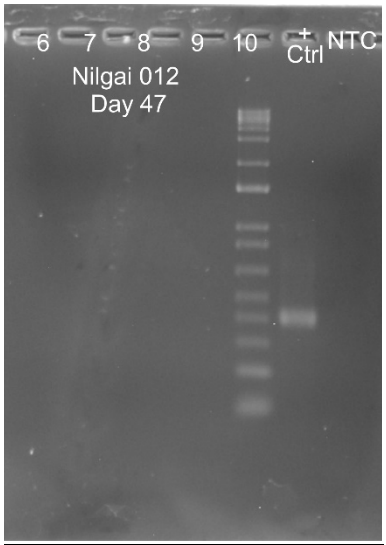
6 7 8 9 10
+
Ctrl
NTC
Nilgai 012
Day 47
